# The Modern View of B Chromosomes Under the Impact of High Scale Omics Analyses

**DOI:** 10.3390/cells8020156

**Published:** 2019-02-13

**Authors:** Syed Farhan Ahmad, Cesar Martins

**Affiliations:** Department of Morphology, Institute of Biosciences at Botucatu, Sao Paulo State University (UNESP), CEP 18618689, Botucatu, SP, Brazil; farhan.phd.unesp@gmail.com

**Keywords:** evolution, cytogenetics, genes, genome, next generation sequencing

## Abstract

Supernumerary B chromosomes (Bs) are extra karyotype units in addition to A chromosomes, and are found in some fungi and thousands of animals and plant species. Bs are uniquely characterized due to their non-Mendelian inheritance, and represent one of the best examples of genomic conflict. Over the last decades, their genetic composition, function and evolution have remained an unresolved query, although a few successful attempts have been made to address these phenomena. A classical concept based on cytogenetics and genetics is that Bs are selfish and abundant with DNA repeats and transposons, and in most cases, they do not carry any function. However, recently, the modern quantum development of high scale multi-omics techniques has shifted B research towards a new-born field that we call “B-omics”. We review the recent literature and add novel perspectives to the B research, discussing the role of new technologies to understand the mechanistic perspectives of the molecular evolution and function of Bs. The modern view states that B chromosomes are enriched with genes for many significant biological functions, including but not limited to the interesting set of genes related to cell cycle and chromosome structure. Furthermore, the presence of B chromosomes could favor genomic rearrangements and influence the nuclear environment affecting the function of other chromatin regions. We hypothesize that B chromosomes might play a key function in driving their transmission and maintenance inside the cell, as well as offer an extra genomic compartment for evolution.

## 1. B Research in the Genomic Era

**Figure 1:**
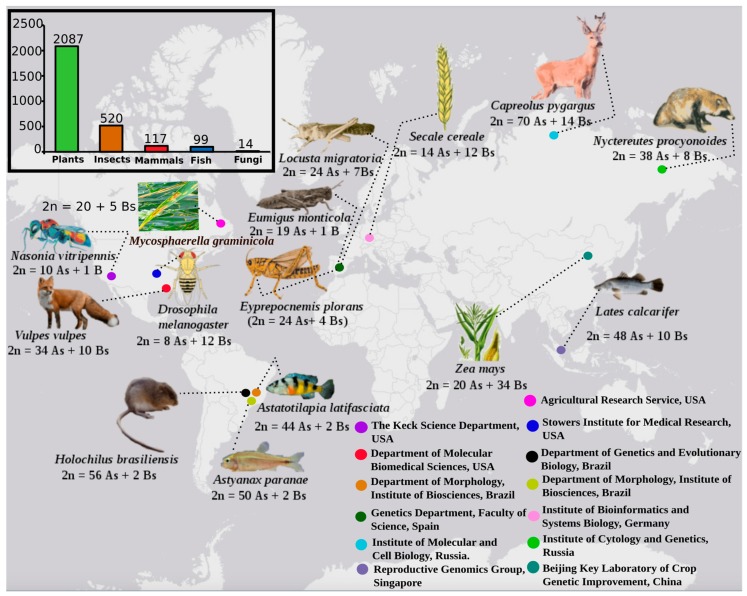
Occurrence of B chromosomes (Bs) in major eukaryotic groups. The bar chart shows the number of B carrier species for different categories, sourced from the B-chrom database [7] (http://www.bchrom.csic.es/). A representation of various commonly used model B carrier species, under the investigation of omics, is shown with karyotype data and information about the geography of research institutes. The revolution of omics-based techniques has gained remarkable attention of B chromosome biologist all over the word working with a huge range of species.

Supernumerary B chromosomes (Bs) are dispensable karyotypic components that show non-Mendelian features and possess a non-standard behavior of inheritance; they lack the ability to undergo recombination/pairing with A chromosomes [1]. The term “B chromosome” was coined for the first time by Randolph in 1928 [2] as a designation for a type of chromosome that differed from the normal chromosomes, which he termed A chromosomes. Although the term B chromosome was introduced by Randolph in 1928 [2], this chromosomal type was firstly described in *Metapedius* true bugs in 1907 [3], and later in 1908 in plant bug insect *Acanthocephala* and in coleopteran insects *Diabrotica soror* and *D. punctata* [4]. It is estimated that Bs occur in about 15% of eukaryotic species [5], and at present, the vast majority of species known to have Bs are plants [6]. Until January 2019, approximately 2087 plants, 744 animals, and 14 fungi species (data based on the B-Chrom database: http://www.bchrom.csic.es/) are currently known to carry these supernumeraries (see the bar chart in Figure 1). But the exact number of B carrying species is unknown [7]. The number is expected to increase with karyotyping and it is believed that there are likely many species possessing Bs that remain unknown at the present time.

The molecular composition of Bs was investigated in the past by classical molecular cytogenetics (mainly chromosome mapping), which provided a milestone for current B chromosomal research. These studies explored repetitive DNA as chromosome probes to discover the localization of specific sequences on Bs. Several repeats of DNA including tandemly arranged repetitive elements [8], LINEs (long interspersed nuclear elements) and SINEs (short interspersed nuclear elements) [9], interstitial telomeric sequences [10,11], ribosomal DNA clusters [12,13], or histone genes [14] were hybridized on Bs. As a result of these studies, it is now known that Bs possess different repetitive elements (also occurring in the standard genome) in all studied species. Further research to discover the molecular structure of Bs involved approaches such as the isolation of B chromosomes by microdissection or flow sorting followed by reverse painting, but these techniques were unsuccessful to advance the concept of homologous regions between A and B chromosomes [15,16,17]. While much has been done to uncover the repeat DNA contents, recent and upcoming exploration of protein coding genes and non-coding RNA sequences on Bs will meet the demands of understanding their enigmatic evolutionary origin.

**Figure 2:**
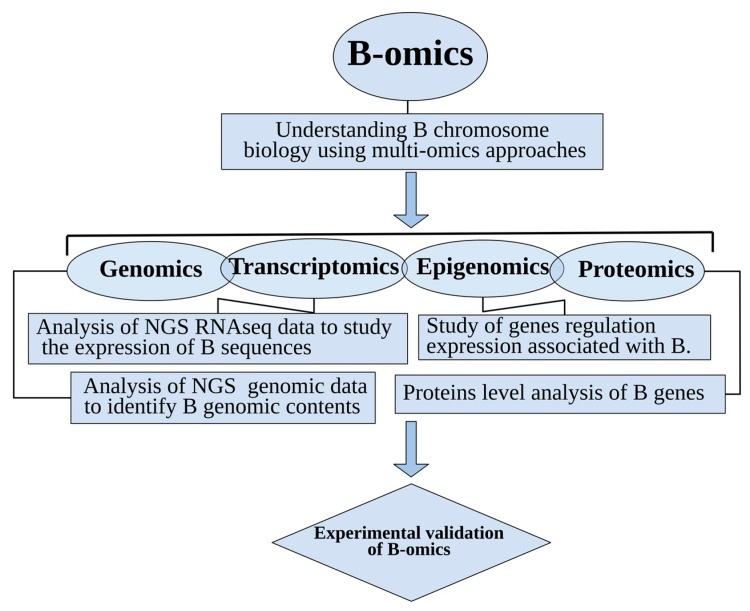
An overview of “B-omics” comprised of multi-omics technologies applied to ongoing research of B chromosomes.

Over the last few years, B chromosomal research has gained the attention of biologists especially in the area of genomics, and the number of research articles about them is on the rise [7,18]. Several perspectives have emerged, investigating B-carrying model species with the help of omics technologies. The most common species widely explored as model organisms for omics analysis applied to B chromosome biology are shown in Figure 1. Next generation sequencing technologies (NGS), along with data analytical approaches, have revolutionized the classical era of B research. The development of recent multi-omics techniques has played a key role in understanding the genomic composition, origin, evolution and biological significance of Bs. These advancements have enabled a novel vision of B-chromosomes and shifted research towards a new-born field that we call “B-omics” (Figure 2). In this review, we highlight the current omics methodologies best applied to identify B sequences, and introduce a new idea for B evolution demonstrating mechanistic concepts of their origin. We review newly published B literature, concerning B-linked genes in different species and discuss the potential approaches applicable to their detection. We also present a modern concept of Bs and provided an updated model demonstrating the possibilities of their evolutionary fate inside the cell. Finally, we describe Bs as an emerging model system to understand genomic underpinnings and address future directions of B chromosomal research.

Box 1 **Glossary.**

**B-blocks:** The putative genomic regions present on B chromosome(s) detected via coverage ratio analysis as a result of NGS read coverage comparison between the two genomes, with and without B(s).

**B chromosomes (Bs):** Chromosomes that are not essential parts of the genome and therefore not present in all individuals in a population of a given species. Most commonly, Bs are named as accessory supernumeraries, additional, extra, or dispensable elements.

**B-omics:** The emerging field of B chromosomal research has applications within multi-omics approaches such as genomics, transcriptomics, epigenomics and proteomics.

**B precursor DNA:** The multi-chromosomal originated DNA which undergo genomic variations and acts as raw source for B formation.

**B-linked genes:** The genes located on the B chromosomes.

**Drive:** A mechanism via which Bs gain their transmission advantage by exceeding the regular (0.5) rate during cell division.

**Duplications:** The amplification of copy numbers of genomic sequences that results in evolutionary novelties in a genome.

**Essential B genes:** B-located genes which are critical in the survival and transmission of Bs during their evolutionary course.

**Proto B:** The primitive B formed during its earliest phases of evolutionary processes. We assume that initial state of proto B might be highly unstable and it might escape through drive to become stable.

**Pseudogenes:** Defined as sequences that resemble known genes but cannot produce a functional protein. However, it is now recognized that they can play role in the regulation of their parent genes or other genes.

**Rearrangements:** The major structural changes in the genome segments, which play a significant role in the evolution of genome structure.

**Transpositions:** The movement of distinct DNA elements (transposons) from one genomic region to another. Transposition is considered as the principal starter event in Bs evolution because of their higher content of transposons.

## 2. Genome Composition, Origin and Evolution of Bs

The evolutionary origin of the B has been under debate for decades. Extensive cytogenetic analysis and the latest B-omics revolution has shown that Bs share sequences with A chromosomes (As). A well-known concept emerged from a series of cytogenetics approaches stated that Bs are derivatives of As [19,20], which was experimentally explained using molecular biology tools in diverse species [21,22,23,24]. High scale genomic analysis has proved to be significantly effective in enabling cytogeneticists to answer questions regarding the molecular mechanisms involved in the evolution and origin of Bs [25]. 

Despite the substantial increase of B chromosome literature reviews [6,26,27,28,29,30,31,32,33,34,35,36,37,38,39,40,41,42] discussing their complex genetics, a deeply understandable concept of their origin is still missing. Although most of these review papers and the latest omics-based research has proposed several models to explain the origin of Bs, these models lack a clear and advanced understanding of the molecular mechanism involved during the formation of Bs. NGS based studies in plants [43], fish [25,44], and mammals [45] have shown that Bs evolved from multiple As (multi-A DNA), as a result of transposition, duplications and rearrangement events. Although B chromosomes have multiple origins in diverse species, they can also originate from a single A chromosome as identified in *Drosophila melanogaster* [37,46]. Although a clear understanding about the origin of the proto-B in *D. melanogaster* is still missing, it is assumed that a centromeric misdivision in chromosome 4 might have triggered the B formation [46]. In other insect species, such as grasshoppers, the Bs are most likely to emerge from different A chromosomes [47,48]. However, Bs origin from the A genome of related species through hybridization processes was reported for plants, fish and insects (for review see reference [26]).

In rye, B sequences contain thousands of genic fragments and some complete genes, which originated as paralogs of As [43]. The Bs of maize [24] and cichlid fishes [25,44] also revealed similar results. The repetitive DNA analysis in *D. melanogaster* shows satellite sequences detected on Bs were also found on As, as well as sex chromosomes [46,49]. In mammals, certain presumptions have been made about origin of Bs based on the results obtained through molecular studies (see review [39]). For instance, in the common echymipera [50], northern collared lemming [51] and small Japanese field mouse [52], sex chromosomes were considered as the major source of the B-chromosome DNA content. In other studies, the Bs of the Korean field mouse showed homology to sex as well as A chromosomes [13,17,53]. The fluorescent in situ hybridization (FISH) and microdissected B sequencing analysis in yellow-necked mice revealed the sequences present on Bs, which were originated from sex chromosomes [54]. The Bs might have a different origin in other mammalian species, for example the Bs of gray brocket deer are homogeneous to the sequences of As [55]. Similarly, the Bs sequences of racoon dogs and red fox revealed similarity to DNA contents of different As, suggesting their multi-chromosomal origin [56]. The comparative genomics analysis in the Bs of fungal species detected an enrichment of transposable elements (TEs), some of which were B specific while others were homologous to As [57,58,59,60]. Many reports dealing with identification of repeat DNA on Bs in various species [61,62,63,64,65,66,67,68] revealed that they are highly enriched with TEs and the data suggests that transposition might have facilitated the initiation of the independent evolution of Bs. The most common and abundant TEs found on Bs of different analyzed species are retrotransposons [25,63,69]. The higher level of retrotransposons can be linked to the formation of Bs because of their ability to mobilize genomic contents from As to B. The activity of these retroelements is considered to play a key role in mediating gene evolution [70,71,72,73].

In addition to repetitive DNA, pseudogene-like sequences have also been reported to be located on Bs [56,74,75]. We assume that these pseudogenes might have formed as a result of intensive rearrangements and frequent sequence duplications in A chromosomes. It has been observed that Bs are expected to occur more frequently mainly in those species that have suffered these chromosomal rearrangements [32]. According to Karamysheva et al. [53], the evolution of Bs in Korean field mice might have occurred in two steps. Firstly, the pericentromeric regions underwent some sort of destabilization followed by accumulation of euchromatic DNA from As, resulting in the birth of proto-Bs known as microchromosomes. Then in the second step, new DNA is inserted and amplified in proto-Bs. A similar model of B evolution was proposed by Rubtsov et al. [76], who hypothesize that the q arm of ancestor As encounter chromosomal rearrangements to make Bs. Another study involving the sequence analysis of Bs in two mammalian species [55] postulated that segmental duplications of certain regions in the genome, subsequently followed by pseudogenization as well as acquisition of various repeats gave rise to the formation of Bs.

**Figure 3:**
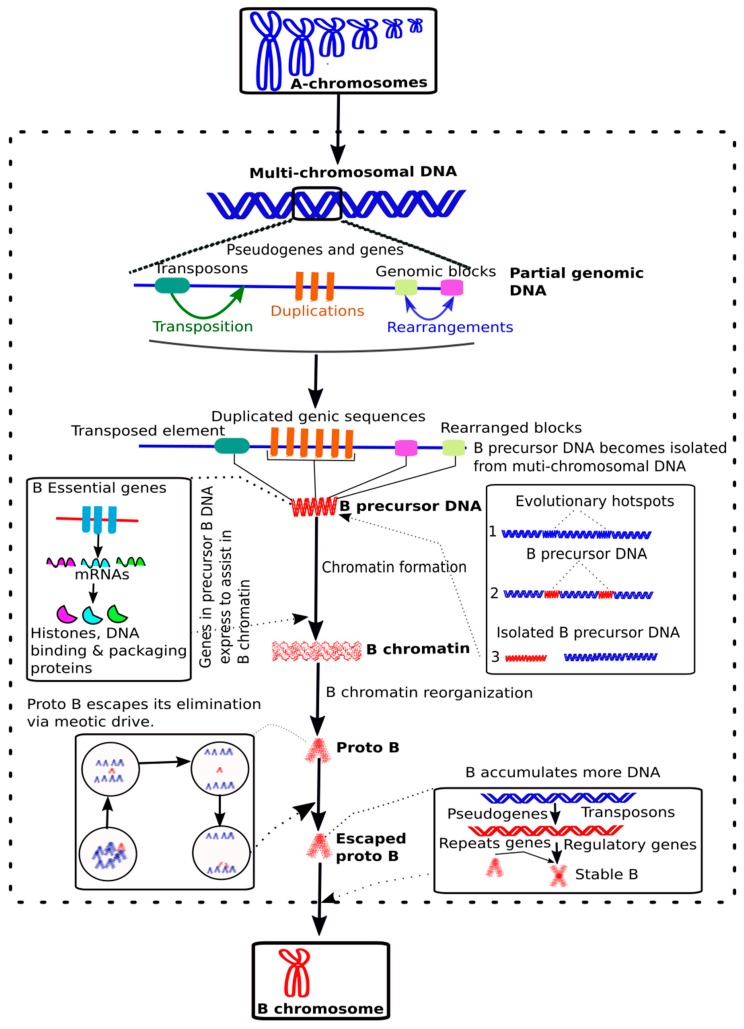
A new model of B evolution. We propose an updated model to illustrate B origin and evolution. Initially, the evolutionary hotspots in multi-chromosomal genomic DNA undergo different events such as transposition, duplications and/or genomic rearrangements; which can be considered as principal evolutionary forces. These events cause the origin of “B precursor DNA” that becomes isolated from the A chromosomes by any genomic rearrangement. The B precursor DNA could contain B essential genes (for example histones, DNA binding and packaging proteins), which are expressed to form B chromatin and its reorganization followed by the accumulation of additional DNA including repeats and genes. This ultimately results in the formation of a nascent proto-B through a series of evolutionary events as depicted. See the topic 2: “Genome composition, origin and evolution of B”, for details.

Apart from the presence of transposons and pseudogenes/fragments of genes on Bs, the latest research studies [25,44,56,75,77,78,79] have also found protein coding intact genes that we call “B essential genes” (see definitions in Box 1) which might be critical for the survival and transmission of Bs during their earlier stages of evolution (see details in next topic section). To create a reference of B-linked genes, we have organized a database that contain a list of genes detected in different species (Supplementary Dataset S1). The studies involving the discoveries of B-linked genes are mainly based upon the NGS analyses of the B contents in different model species that have provided a better picture of B ancestry. Based in the knowledge extracted from B-omics, it can be hypothesized that the occurrence of a series of events like transposition, frequent sequence duplications and multiple genomic rearrangements in the donor As might have favored the formation of a “B-precursor DNA” (Figure 3). This B-precursor DNA might possibly be enriched with TEs, other repeats, pseudogenes fragments and intact B essential genes. The abundance of retrotransposons in B-precursor DNA might have caused the mobilization of B essential genes from different As. Initially, the B-precursor DNA could have isolated itself from A-genomic DNA by any evolutionary force acting over A chromosome rearrangements, resulting in independent small chromosome fragments. An analogy of such a mechanism has been proposed for the evolution of younger genomic elements, such as the small supernumerary marker chromosomes (sSMC) in humans [80]. Sequencing based analysis of sSMCs and Bs genomic content have been reported [56,80] providing insights into their origin. During its initial independent life, the B-precursor chromosome could be highly unstable like sSMCs. The B essential genes in B-precursor DNA might have transcribed RNAs and encoded proteins that assist in B chromatin organization and maintenance. The B-chromatin acted as a primary source for forming a proto-B that could have gone through an escape process during the cell cycle by drive, as has extensively described for Bs [81]. Because of the relaxed selective pressure on genic sequences and rapidly evolving DNA repeats of newly formed Bs, this might benefit the acquisition of further structural changes that could facilitate the drive during the cell cycle. The escaped proto-B then might have accumulated many DNA sequences during its evolutionary course to become a stable mature B through the aforementioned mechanism.

## 3. Discovering B Genes: The Current Arena for B-Omics

As a result of updated information about the genomic contents of Bs, it is now inferred that B chromosomes, once considered as entirely heterochromatic and genetically inert, are also constituted by processed pseudogenes, non-coding RNA transcribing sequences and protein-coding genes. The latest omics techniques have been applied to study Bs and empowered researchers to interpret their evolutionary origin and complex composition. The revelation of numerous multiple A chromosomal genes on Bs started a new debate about their evolutionary role, their complex interactions with the host genome and their possible effects ranging from sex determination to fitness and adaptation.

A couple of reviews [31,32] have reported the gene contents previously identified on Bs with limitations of focusing only on specific groups of genes and species. Here, we briefly revise the summary of previous and latest published papers on B-linked genes and provide an updated comprehensive list of hitherto detected B genes in diverse species (see the genes listed in Table 1 and Supplementary Dataset S1). Cytogenetics based studies have found functional sequences on Bs, focused on locating rRNA genes, which were easier to detect because of their high copy number (see review, [82]). These rRNA genes have been successfully identified on the Bs of *Secale cereale* [83], *Brachyscome dichromosomatica* [84], *Crepis capillaris* [85], *Trichogramma kaykai* [86], *Eyprepocnemis plorans* [87] and *Astatotilapia latifasciata* [88]. Many studies have reported snRNA and histone genes in the Bs of different species [14,40,89,90,91,92]. The Bs of oat (*Avena saliva*) carrying transcriptionally active genes show effects on host resistance to rust [93]. An antibiotics resistance study of the fungus, *Nectria haematococca*, revealed the presence of genes on supernumerary chromosomes for the first time [94,95]. This was followed by another study that further led to characterization of a gene on the extra karyotype elements with evidence for expression [96]. The cytogenetics analysis of racoon dogs revealed that Bs are rich in sequences such as inactive copies of ribosomal genes and interstitial telomeric sequences [10,11]. Other studies [45,97] revealed the existence of a B-localized C-KIT gene in the mammals *Nyctereutes procyonoides* and *Vulpes vulpes*. Another analysis [98] revealed the first transcribing vertebrate genes occurring on Bs in the Siberian roe deer (*Capreolus pygargus*). These genes, namely TNNI3K, FPGT and LRRIQ3, comprised a 2 Mbp region, and were shown to be unevenly amplified among Bs of Siberian roe deer.

During the postgenomic era, bioinformatics and genomics tools have caused a sudden acceleration in revealing B-located genes. At the beginning, the genome sequencing project of *N. haematococca* showed that Bs carried genes that are involved in expansion of the ecological niche of the host [65]. Direct sequencing of Bs isolated from the rest of the genome via microdissection and flow sorting techniques in plants (rye) enabled researchers to find thousands of genic sequences residing in Bs [43]. An example of B-linked genes is illustrated from the study of B-carrying *Lithochromis rubripinnis* cichlid fish [99]. This study involved the identification of B-specific clones based on differential hybridization with genomic DNA of individuals with B (called B+) and without B (called B-) chromosomes. This analysis identified a B-specific repeat and fragments of five protein-coding genes. One of the genes, IHHB, was extensively amplified and about 40 copies were found in the *L. rubripinnis* B+ genome [99]. Remarkable progress was made during a study aimed to investigate the origin and evolution of B chromosomes in *A. latifasciata*, a cichlid species, using a whole genome sequencing approach. Several B-linked genes were reported, involved in chromosomal segregation: Proteins involved in microtubule organization (TUBB1, TUBB5), kinetochore structure (SKA1, KIF11, CENP-E), recombination (XRCC2, SYCP2, RTEL1) and progression through the cell cycle (Separase, AURK) [25]. Some of these B-linked genes were found to be expressed in the related cichlid species *Pundamilia nyererei*. The genome analysis of *Drosophila albomicans* identified active B-linked genes [100]. Recent studies rely upon integrative genomics and transcriptomics approaches to identify the B-linked genes and check their expression levels. For example, in *Nasonia vitripennis*, a transcriptomics-based study of a B-like element, a so called paternal sex ratio (PSR), detected a total of nine transcripts located in the PSR [101]. Yet another new example is the genomic and transcriptomic analysis applied to rye B that revealed B-located pseudogenic sequences and active copies of an Argonaute-like AGO4B gene [77]. More recently, a combination of comparative genomics and transcriptomics analysis of B- and B+ individuals of *E. plorans*, found a total of four intact and six fragmented genes localized on Bs [78]. The expression activity of these identified genes were traced by the relative abundance of transcripts between B+ and B- samples. The researchers discovered that five of these genes, namely CIP2A, CKAP2, CAP-G, KIF20A, and MYCB2 were remarkably upregulated and actively transcribed in B+ organisms. Another new paper [102] identified protein coding genic regions on the B chromosome of *Zea mays*. They identified upregulated genes on the B chromosome, which are mainly involved in cell metabolism and nucleotide binding. According to this study, the most significant terms in biological processes detected from Gene Ontology (GO) analysis in the B chromosome of maize are cellular processes and metabolic processes, while that of molecular functions showed most of the genes being involved in ribonucleotide and deoxyribonucleotide binding. The sequencing analysis of flow sorted Bs in the reptilian species *Anolis carolinensis* detected cell division associated genes, namely INCENP and SPIRE2 [75]. The characterization of B sequences in Chinese racoon dog and red fox have discovered B-located genes encoding functions for development, cell cycle and neuron synapses [55]. The integrative Illumina and Pacbio sequencing data analysis of several cichlids species from Lake Malawi identified many gene fragments and genes located on Bs, some of which could possibly function in the regulation of Bs establishment [44]. The sequences of microdissected Bs in a fish species, Asian Seabass, were assembled and their annotations recorded a total of 75 genes out of which 10 genes, including ASTN2, BRE, DPF3, FBXO33, FKBP, GABRB2, MYT1L, RAB14, RXRAB, and PACRG, were known to be involved with particular expression in the brain and gonads [79].

**Figure 4:**
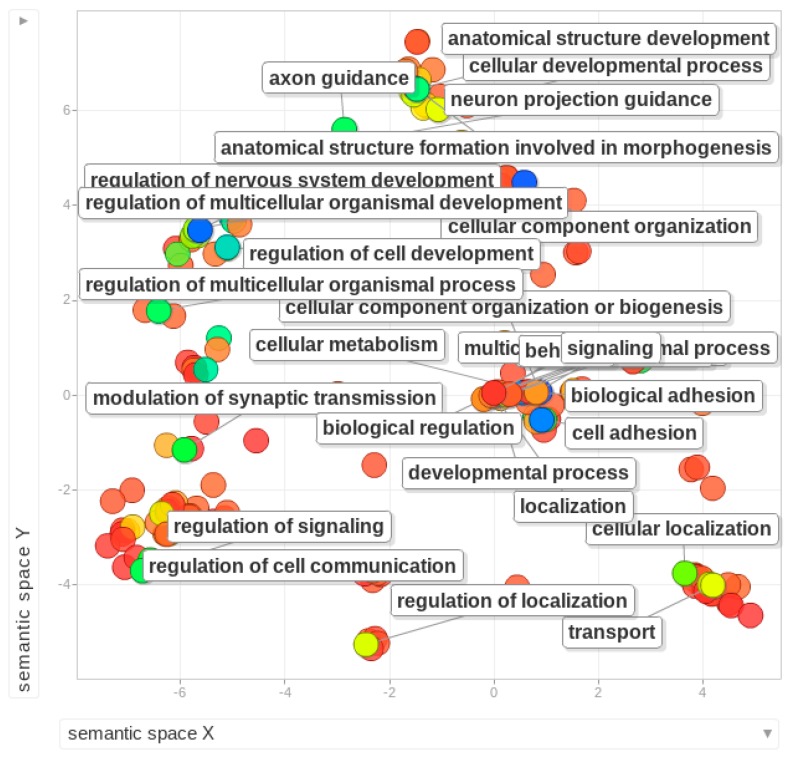
**Gene ontology** (GO) enrichment analysis of B-linked genes reported in diverse species [25,44,48,79]. Enriched terms are shown as the bubble plot using human as reference database. Different colors of bubbles show the intensity of enrichment for labeled function based on the log10 of P values, ranging from dark blue with highest level of enrichment. The X and Y axis do not have any intrinsic meaning.

More recent analysis of 0B and 1B transcriptomes in *E. plorans* has shown the presence of Bs causes differential expression of 46 genes associated with different functions such as cell death, histone-methyltransferase activity, protein modification, regulation of genes, stress response and chemical defense [107]. Apart from the listed genes in Table 1, we gathered an extensive list of genes and gene fragments (a total of 17,139) that have been reported on the Bs of different species [25,44,50,79,107]. This list is given as Supplementary Dataset S1 and can be used as a reference database for further investigation of B-linked genes. We also searched these genes in the Ensembl database (https://useast.ensembl.org/index.html) and retrieved their Ensemble IDs with respective descriptions (Supplementary Dataset S2). We performed a GO enrichment analysis of these genes to check what functions are commonly enriched among the Bs of diverse species (Figure 4). Because the list contains a mixture of genes from different species, mostly fishes and mammals, two different enrichment analyses were performed using human and zebrafish as reference databases. The enriched terms included a set of different important biological processes such as cellular process development, cell adhesions, nerve system, immune response, localizations, morphogenesis, regulation of gene expression, metabolism and chromosome segregation (Figure 4). The groups of enriched genes with functions, GO and statistics (using human database as a reference) are listed as Supplementary Dataset S3. The molecular functions and cellular component GO categories resulted in enriched terms such as protein binding, cell parts and synapse and cell junctions. GO enrichment was performed using the Gorilla tool [108] and visualized by Revigo [109].

## 4. Genomics Strategies for Efficient Gene Hunting on Bs

**Figure 5:**
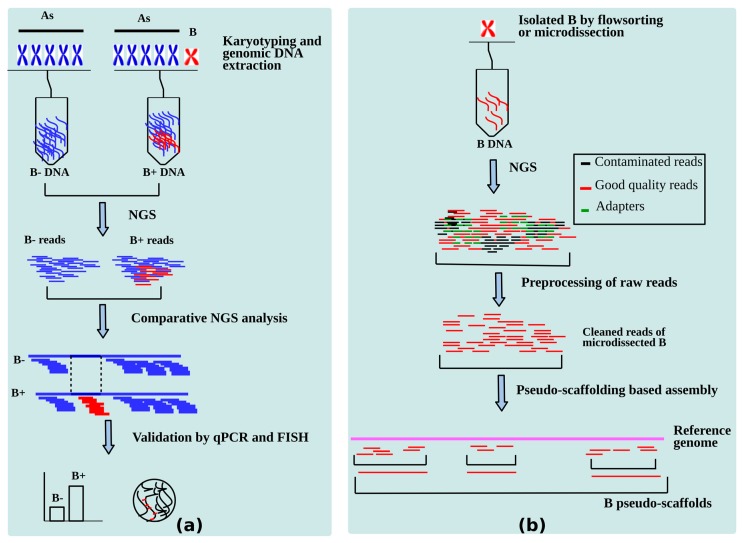
NGS based methods for identification of B-linked genes. (**a**) A Summary of comparative genomics of samples with B chromosomes (B+) and without B chromosome (B-) steps for identification of putative sequences on B. (**b**) Genomics analysis of microdissected/flow sorted B.

Identification of genes is the primary step in understanding the genome biology of an organism after performing its whole genome sequencing [110]. The current arena for B-chromosome research is the gene identification in Bs, which is increasing in pace to uncover their function. As discussed in the last section, for the last decade or so, a number of B carrying sequenced genome studies have focused on developing analytics for B-linked gene hunts. A summary of the applied methodologies that have been successfully established to mining B-located genes is presented in Figure 5. The sequences present on Bs are identified by NGS analysis followed by validation by FISH and quantitative PCR (qPCR) experiments and then the biological role of these sequences can be narrowed.

Contemporary sequencing technologies and state of art bioinformatics has great potential to explore the genomic composition of Bs. The combination of NGS and modern bioinformatics approaches has provided exciting information to identify B-located sequences and study their role. This high scale genomic analysis can be achieved by the strategies developed on the bases of two subsections of genomics, i.e., compositional genomics and functional genomics. The compositional genomics of Bs constitute the approaches using NGS data that are aimed at detection of genic and repeat sequences. Ruban et al. [105], have summarized this in silico analysis in two principal strategies, i.e., direct and indirect methods. The direct method involves the NGS analysis of the isolated (flow sorted or microdissected) Bs. While the indirect method comprises of comparison of NGS data between the genomes with and without B(s) with the application of three alternative ways namely “similarity-based read clustering”, “coverage ratio analysis” and “kmer frequency ratio analysis”. Once the B-located sequences are detected, then there comes the next step of understanding their biological role. This is performed by functional annotation, which includes a series of steps such as comparing the B-sequences with a reference gene set of closely related species, repeat finding, aligning the genic sequences with protein sets of the closely related reference species, calculating the gene integrity, mapping of annotated genes with GO database and the enrichment analysis of ontologies. The proposed methodologies of annotations are mainly based upon the methods adopted by Valente et al. [25].

The newer approaches applied to the detection of B-linked genes in cichlids [44] were also based upon a similar methodology. Thousands of B-blocks were found by calculating the coverage ratio of the aligned B+ and B- reads to the reference genome and the significant differences in the read coverage were detected by statistical analyses. Another procedure for identification of protein coding genes was employed to *E. plorans* Bs by Navarro-Domínguez et al. [78]. In this procedure, the B+ and B- reads were aligned to a reference transcriptome assembly and the abundance of mapped reads were counted for all coding sequences (CDS). Then, a log_(2)_ of B+/B- was calculated for all mapped reads against CDS and the values obtained greater than the threshold (set on the basis of gene copies on As and Bs) were extracted and assumed to be present on the B chromosome. The copies of B-linked genes were then validated by qPCR.

Considering some limitations to the aforementioned strategies applied to Bs analysis, we propose a novel approach that could enable researchers to identify and understand the sequence contents on Bs in a more efficient way. Some of the drawbacks in the established methodologies discussed above are: (1) Although the microdissection of Bs is a relatively precise way to identify the B-linked sequences, this technique needs an amplification step before sequencing and the sequences can get contaminated or over represented. (2) The flow-sorting technique, which involves the separation of a chromosome using laser charges, is based upon its size and GC contents, in most cases they are sorted with other chromosomes that have similar GC content. Also, this technique is not useful for small chromosomes (a majority of Bs are smaller than As) because of frequent sorting with debris of larger chromosomes. (3) The detection of B-blocks on the basis of coverage ratio might not be completely accurate and could yield many false positive regions, resulting in the over representation of the amount of genes present on Bs. 

**Figure 6:**
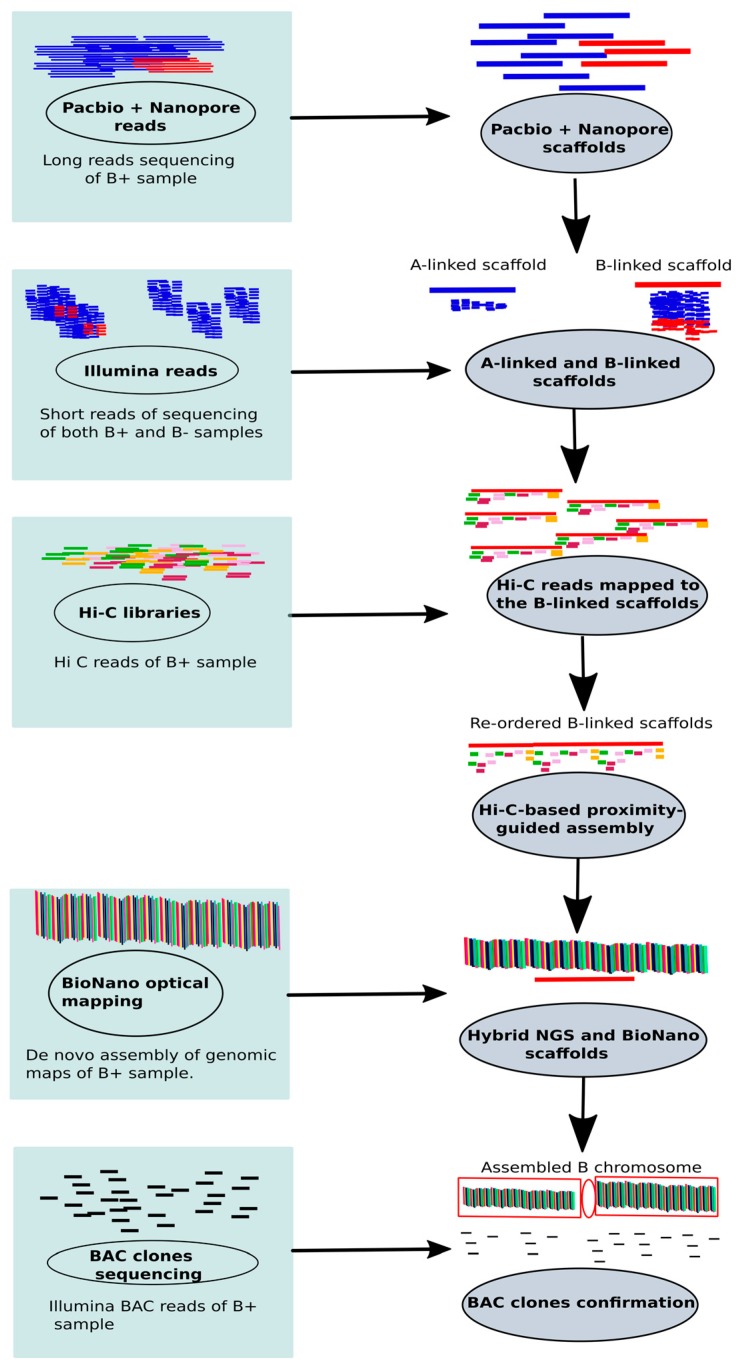
An illustration of a combination of different technologies applied to achieve an accurate and error free assembly of B chromosomes. Long PacBio and Nanopore sequencing reads are generated for the B+ sample. The long reads are assembled producing a mixture of A chromosome and B-linked scaffolds. To separate the B-linked scaffolds from the rest of genome, Illumina short B- and B+ reads are mapped to this assembly. The mapped reads are counted for each scaffold and a coverage ratio for both B- and B+ is calculated. The B-linked scaffolds are isolated with the higher coverage of B+ than B- reads. Then, Hi-C libraries are created for B+ sample and mapped against the identified B-linked scaffolds. The scaffolds are merged into a single molecule now called the B-assembled chromosome utilizing the order and orientation information from Hi-C data. The BioNano assembly of B+ sample is performed and the hybrid maps are generated from the BioNano scaffolds and the B-assembled chromosome. The B chromosome assembly is validated by alignments of bacterial artificial chromosome (BAC) clone sequences and physical chromosome mapping confirmation based on the use of BAC clones as FISH probes. This step involves the process of identifying and correcting the errors with BAC clones spanning scaffolded B chromosome. The illustrated proposed methodology has been successfully applied to sex chromosome assembly. For more details refer to the methodology section of the article Mahajan et al. [112].

To address these challenges, we put forward the idea of assembling B chromosomes utilizing a combination of modern technologies such as long reads (PacBio and Oxford Nanopore) sequencing, BioNano (Phase genomics) and chromatin conformation capture (Hi-C) libraries. The PacBio sequencing in cichlid fish has revealed the genomic contents of Bs [44]. Inspired from the sex chromosome assemblies (see review, [111]), we present a new methodology to recover the entire B with the highest accuracy. The steps of our proposed method are summarized in Figure 6. This method is principally based on the steps recently applied successfully to assemble the Y chromosome of *Drosophila* (see details in the methods section of the paper, Mahajan et al. [112]). Because of the similarity between the B and sex chromosomes, in the context that both chromosomes have smaller sizes and are difficult to assemble due to the higher repeat content, we propose that this method can potentially resolve the problems of Bs analysis. 

## 5. B Genes Evolution. A Parasitic Versus Non-Parasitic Scenario

The most common genes reported in the Bs of different organisms encode for cell cycle and chromosomes related functions (Table 1; Box 2). There are also other interesting sets of B-linked genes that may involve important biological processes including metabolism, regulation, transport, development, nervous system, or sexual functions. Genes with these kinds of functions were reported in previous studies to be located on B chromosomes [25,27,78,81]. Bs were also reported to influence the genes on the A chromosomes [34,107]. This may suggest that Bs, which at one stage were considered selfish parasites during their evolutionary life, seem to be turning out to be regular elements after gaining stability. It seems that Bs originated as a small chromosome/genome fragment that get stabilized after successful escape from the cell cycle control, i.e., they do not get eliminated during cell division and are transmitted to the next generation. These genes are momentous to the initial survival of the proto-B. The proto-B then gains or contains cell cycle genes and can take advantage of cell division to escape elimination. This ability gives the selfishness/parasitic characteristic to Bs. After the B is stabilized, it is free of the strong mechanism of cell division control and continues getting new sequences (Figure 3). This mechanism hints that the B is like a “repository” of gene/genomic variants that can be co-opted by the regular genome at any time. This means that it is no longer only a selfish element, but can also contribute to the whole genome. Probably that is the reason why Bs have been connected with effects covering diverse important biological processes.

Box 2 **Bs are more than selfish elements.**

An interesting concern arising from the ongoing increase in the discovery of B-linked genes and their functions over the whole nucleus environment (as reported in a number of studies [25,44,75,78] is “why Bs are so enriched with genes involved in the functions related to important cell processes?”. Although the mechanism by which they accumulated these genes is unknown, the reason for possessing such types of genes can be their selfish nature, as described by many researchers. The latest data provides a list of B genes that, we speculate, might have played a role in the Bs formation. Some of these genes, which are intact, encode important functions such as cell division and chromosome structure. It is highly expected that Bs derived these genes for their own advantage, which might have played their part in the persistence of unpaired Bs during the cell cycle. We argue that right after the birth of a proto-B, it might have struggled through the hard time of its maintenance, thus evolving chromosome drive mechanisms by the gain of these genes to become a stable B. Because, these genes are critical in shaping the evolutionary process and survival of Bs, we therefore called them “essential B genes”. The genes on Bs reported by recent published studies in variety of species, encoding common functions like cell cycle and chromosome related processes, indicates a similar sort of mechanism of Bs evolution. Thus, the findings of shared B-linked genes across species of different origins will further provide important clue about their origin and emergence.

Bs were often regarded as completely parasitic or selfish karyotype elements that were supposed to seize control of cell system necessary for their replication and successful transmission [19,26,113]. Some studies reported them to be genetically inert while others described them as junk products of As due to their heterochromatic composition. Recently, this scenario has been changed as evidence continues to grow finding many functional genes on Bs. The findings of transcriptionally active B genes disprove the “B genetic inertness” hypothesis; however, complete evidence of whether these genes actually generate proteins is missing [78]. The latest studies revealed some intact genes, which are exceptions to the specific functions required for their own evolution or maintenance. Instead, these genes are related to several other important roles such as sex determination, metabolism, development, nervous system, ion transport, among others. Remarkably, these genes include those that are essential in gene regulation processes. This suggests that Bs tend to become an essential part of the genome. Ongoing transcriptomic and epigenetic analyses are revealing the effects of B sequences in the expression pattern of genes on As. Thus, it can be hypothesized that the final destiny of the stabilized Bs might be a shift from selfish to unselfish, contributing its reorganized DNA material to the species.

In the context of B-linked genes, we suppose that the successful evolution of these genes might play a significant role in deciding the successful evolutionary fate of Bs resulting in the ultimate disappearance or establishment of Bs. The classical view of Bs (presented by Camacho et al. [26]) has predicted a parasitic mechanism describing the life cycle of a B. According to the Camacho model, the newly formed B is highly unstable and its parasitic drive ability suffers suppression because of neutralization effects triggered by the evolution of certain A chromosome genes. The neutralization of the B parasitic “drive” results in a random walk of the B chromosome leading towards its extinction. Earlier studies [19,114,115,116,117,118,119] had hypothesized that A genes tend to neutralize the parasitic effect of Bs presence by some variations in order to reduce the damage caused to their carriers. 

**Figure 7:**
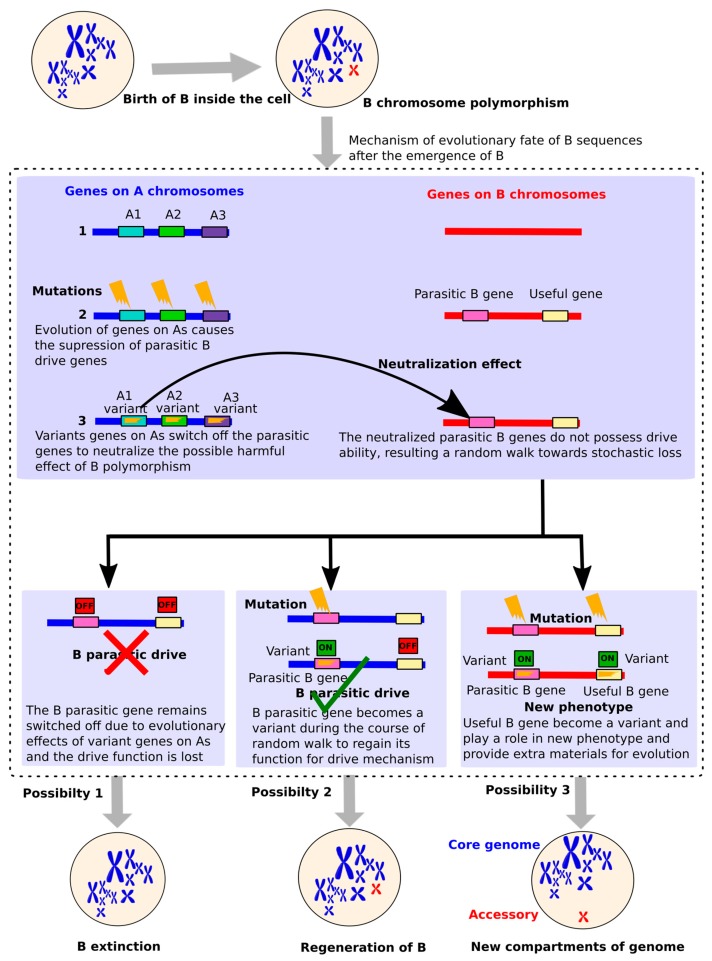
Evolutionary fate of the B in the light of gene evolution. After the B chromosome polymorphism appears, certain genes in A genomes suppress the B parasitic genes that are required for the drive of B during cell cycle transmission. These A genes neutralize the harmfulness of the parasitic drive by switching off the B genes because of mutations induced during their evolution. This mechanism might output three different possibilities: (1) B genes might not experience any mutations and remain switched off due to which the drive is completely lost resulting in the B extinction; (2) the B parasitic genes undergo some mutations to escape from neutralization effects and thus regain their functions so that drive is maintained leading to the regeneration of the B; or (3) both parasitic and useful genes present on B might experience mutations so that parasitic genes regain their function and ensure the regeneration of the B while useful genes might also be expressed, resulting in the formation of a new phenotype. The third scenario is evidenced from the B-linked genes found in fungi (see review, [36]), in which these genes might encounter selection to useful phenotype. This is achieved as a result of compartmentalization of the genome into core and accessories parts where the later part can serve as extra material for adaptive evolution.

The suppressed drive might, however, be regained by some mutations in B causing its regeneration. Based upon the growing amount of data supporting the presence of genes with unselfish functions, we proposed an updated model to give an evolutionary idea about the selfish (parasitic), unselfish (non-parasitic) and/or useful nature of B-linked genes (Figure 7). We, with the support of newly published data, update the previous model about the parasitic nature and reflect a new picture of Bs evolution.

## 6. Bs: An Exciting Model System to Study Genome Evolution

Bs have become one of the major foci in chromosome biology studies because they offer an excellent model to investigate mechanisms of chromosome changes during evolution that, in some cases, are incipient. Although several factors that mediate genomic changes are now known, there are still many unanswered questions. In light of the high throughput era of generation of biological data, chromosome studies have also advanced to a higher level exploring high scale analyses of DNA, RNA, and proteins, and their interactive networks [34]. The leading role of NGS and bioinformatics methods applied to Bs is capable to advance our understanding on genome dynamics such as the evolution of genomes and gene regulations [120]. Bs have emerged as a special model system due to the reason that they possess several unique traits compared to the other chromosomes of the genome related to origin, composition, and evolution, and are characterized as “odd” elements in relation to the rest of the genome. Future analysis using Bs as models will shed light on the mechanisms involved in its rapid genome changes [105] as well as the possibility of being utilized as experimental tools in cancer research (see review, [121]), manipulation of crops, construction of artificial chromosome (see review, [6]), and many other contributions to exciting areas of genome biology. Below, we focus on the emerging role of Bs as model system to understand some important underpinnings of major events that happen during genome evolution.

### 6.1. Bs as Tools to Review the Polymorphism and Structural Variations in Genome

Genetic polymorphisms are natural events that cause structural variations (SVs) in the genome and are considered as major evolutionary forces. Generally, SVs are the rearrangements of genomic regions resulting because of various DNA level mutations such as insertions, deletions, copy number variations (duplications), translocations and inversions. Genetic polymorphism may also include other forms of DNA variations e.g., single nucleotide insertions or deletions (indels) and single nucleotide polymorphisms (SNPs) [122]. The NGS era has exceptionally provided us with a detailed vision of these evolutionary forces that are key players in shaping chromosomal structure. Genomics techniques and associated computer tools are now in action to unveil a variety of rearrangement events that are hotspots for chromosomal evolution [123]. How can Bs contribute to advancing our knowledge in this area? We now know that Bs, as compared to As, are extraordinarily rapidly evolving genomic elements. Bs can be an excellent model system to study localized evolution of genomes and can be achieved by comparing A and B sequences, revealing genomic regions with patterns of chromosomal rearrangements. The research using Bs as a model can help elevate our understanding about the mechanisms that can shape the formation of new chromosomes during the evolutionary course of a genome. Although the latest studies [24,25,43,103] involving large scale genome analysis of polymorphisms and variations of B sequences have enabled us to predict the process of formation and to trace the evolutionary history of Bs, our knowledge of how genomic variations cause their evolution is still limited. Chromosome rearrangements are triggers of evolutionary changes and are also of huge importance in disease origins. Bs represent exciting models to investigate chromosome changes because they occur in populations as post-rearranged structures and it is important to find out the molecular basis that govern the chromosomal rearrangements that occurred during their evolutionary processes. In addition, further research using Bs as a model system will principally play a significant role to identify fast evolving lineage-specific gene families. We propose that comparative analysis of genomes with and without Bs based on genomics, transcriptomics, epigenomics and proteomics data, will bring novel insights to understand how the chromosome/genome changes have acted over evolution and over the gain of new genomic functions.

### 6.2. Bs Applications to Explore Genome Architecture

As discussed earlier, the unique characteristics of Bs make them amenable to be used as a model for different genomic studies, for which standard As are inappropriate. The previous extensive cytogenetics research of Bs (see review, [34]) has contributed to the knowledge on chromosomes/genome organizations and provided a platform to direct further studies in order to understand genome architecture. Investigating how the genome architecture and chromatin organizations play their important role in cellular processes are the major concerns of cell biology [124]. The rapid increase in whole genome sequencing projects has fostered a comparative genomics approache through which the high-level exploration of genome architectural features became possible. Such work has unlocked the molecular mechanisms of evolutionary events such as genomic rearrangements, whole genome duplications, and different types of recombinations [125]. We speculate that comparative genomics applied to Bs, in which genomes with only As are compared to the genome with As plus Bs, have great potential in future research in this area. One of the exciting applications of comparative genomics is to identify syntenic regions (genomic regions that are conserved and share the same ancestry) that can provide clues about the genome structure and mechanism of evolution [126]. Bs are more prone to rearrangements and other genomic changes due to their rapidly evolving nature and their comparative genomics make them efficient model candidates to identify syntenies. Comparative analysis of genomes with and without Bs can offer an exciting area of future research that will allow deep insights to study the overall structure, organization and architecture of the genome. Such comparisons will finally reinforce knowledge about the differences and similarities between genomes and improve further understanding about the genomic composition, organization of genes and syntenic regions.

### 6.3. Bs Research Contribution in Understanding Regulatory Mechanisms of Coding and Non-Coding Genome

It is known that only a small percent of the genome comprise sequences that code for proteins while the remaining majority portion is non-coding and sometime called junk DNA [127]. The non-coding regions are transposable elements, structural variants, segmental duplications, simple and tandem repeats, conserved noncoding elements, functional noncoding RNAs, regulatory elements, and pseudogenes [128]. The non-regulatory sequences can play a role in the expression of the coding parts [129]. 

The latest research involving functional annotations and compositional genomics strategies of Bs has facilitated the interpretation of the coding and non-coding genome (see review, [18]). The progress in gene findings on Bs have characterized different concerns about the regulation of gene expression. For example, the activity of B-located genes might affect the expression pattern of genes on As through non-coding RNAs [102,130,131]. Bs can be used as excellent tools in epigenetic research to study the connection between coding and non-coding sequences. The detection of non-coding RNAs on Bs and their in-depth analysis can further foster an understanding of complex questions being asked in modern RNA biology.

## 7. Future Directions

As pointed out earlier, the latest research on Bs is providing remarkable advances in different areas of genome biology. High scale compositional genomics analysis is considered as the primary step because of its great potential to unveil the genomic contents of Bs. The knowledge about the sequence composition can offer possible explanations of their evolutionary origin and further makes way for in-depth functional analysis based on transcriptomics, epigenomics and proteomics. The research on Bs has been further fostered with the recent use of NGS technologies. Although a complete understanding about their role in the genome and evolutionary success still remains a mystery, the hitherto performed integrative analyses have contributed significantly to the exciting discoveries about the origin, evolution and composition of Bs, (see a Summary in Box 3). In the future, a comprehensive understanding to reveal the complex history of Bs can be possible with the application of multi omics approaches. The ongoing trancriptomics analysis of B chromosomes has shown changes in the expression level of mRNAs and non-coding RNAs, thus reinforcing the idea that B chromosomes should directly or indirectly affect complex regulatory mechanisms in the cell. The large-scale analysis of Bs related transcripts using epigenomics approaches, such as investigating miRNAs and lncRNAs, will help in understanding these mechanisms. The emergence of knowledge around the genomic composition, function and epigenetic regulation of Bs would be necessary to study these issues at the protein level. This analysis involving proteomics coupled with systems biology will further elucidate the influence of B chromosomes in specific cellular pathways to provide a better glimpse of understudied biological networks. In summary, the emerging field of B-omics will surely enhance our understanding about the currently unknown phenomenon of B genomic content and evolution in particular, as well as the genome studies in general. In conclusion, the recent ongoing exploration of sequencing data of Bs has turned these chromosomes into an exciting model system for genome and chromosome biology studies.

Box 3 **Summary: Highlights of Bs research.**

What we know about Bs? The hitherto accomplished studies have deciphered some remarkable discoveries about Bs. Taken together, these discovered features from the rapidly increasing literature in the last century, a theory, which we called “The B chromosomal theory”, can be postulated as:

1. Mostly, the presence of Bs do not affect the phenotype; however, there are a few exceptions.
2. Bs do not pair with As and do not obey Mendelian laws.
3. Bs survive themselves during the cell cycle by drive, although the molecular mechanism is poorly understood.
4. Bs generally are highly enriched in repetitive and selfish DNA.
5. Bs have a multi-chromosomal origin, i.e., they are derivatives of duplicated sequences sourced from multiple As and some cases with additional organellar DNA.
6. Bs possess thousands of fragmented genic sequences and a few complete genes. The completed genes are considered key players in Bs evolution and can also influence diverse phenotypes.

What we do not know about Bs? The above concluding remarks presented as “The B chromosomal theory” has provided an essential knowledge base from which several questions arise to be addressed by ongoing and upcoming research. The principal goals of these questions currently being investigated are:

1. A complete understanding of evolutionary forces that might have triggered the formation of Bs: There are reports which speculate transpositions, duplications and rearrangements as the principal events (as proposed in our model in Figure 3) acting over B evolution, the molecular mechanisms are still not clearly understood.
2. The mechanistic molecular basis of the chromosome drive. This analysis will be crucial to infer how Bs maintenance and survival occurs inside the cell.
3. Regulatory role of B-located genic sequences. The latest works have provided a preliminary view that B genes might have effects on the pattern and level of expression of A-located genes. Thus, a detailed examination of the epigenetic status to understand these effects is required.
4. A better picture of the sequence composition and organization of the B genome. As pointed out earlier, strategies have been developed to reveal their genomic content, however more accurate analysis is essential. This can possibly be achieved by obtaining high coverage NGS sequences based on new sequencing platforms followed by complete chromosome level assembly of Bs as well as chromatin conformation studies.

## Figures and Tables

**Table 1:** A comprehensive list of genes previously detected on Bs of different species. An additional extensive list of genes (thousands in number) is given as Supplementary Dataset S1.

Gene	Organism	Gene Function	Reference
Proto-oncogene C-KIT	*V. vulpes* *N. procynoides*	Proto-oncogene, encoding a type 3 transmembrane receptor	[55,56,97]
LRP1B (Low density lipoprotein receptor-related protein 1B)	*V. vulpes*	Cell process of receptor-mediated endocytosis	[56]
CTNND2 (Cadherin-associated protein)	*V. vulpes*	Neuronal cell adhesion and tissue morphogenesis	[56]
45S ribosomal RNA	*S. cereale* *B. dichromosomatica* *C. capillaris* *T. kaykai* *E. plorans* *A. latifasciata*	Protein formation	[83,84,85,86,87,91,103]
FHIT (Fragile histidine triad)	*A. flavicollis*	Tumor suppressor and protein binding and hydrolase activity	[104]
CCT6B (Chaperonin containing TCP1 subunit 6B)	*A. flavicollis*	Folding of actin and tubulin	[104]
TCP-1 (T-complex protein 1)	*A. flavicollis*	Folding of actin and tubulin	[104]
KDR (Kinase insert domain receptor)	*N. procynoides*	Angiogenesis, vascular development, vascular permeability, and embryonic hematopoiesis	[56]
H3 and H4 histones	*L. migratoria* *A. flavolineata*	Transcription regulation, DNA repair, DNA replication and chromosomal stability	[14,90]
RET (Ret proto-oncogene)	*N. procynoides*	Protooncogene, encoding a tyrosine kinase receptor	[56]
LRIG1 (Leucine rich repeats and immunoglobulin like Domains 1)	*N. procynoides*	Negative regulator of signaling by receptor tyrosine kinases	[56]
IHHB (Indian hedgehog b)	*L. rubripinnis* *A. latifasciata*	Developmental processes including growth, patterning and morphogenesis	[99,103]
Ryanodine receptor–like unnamed protein	*Tetraodon nigroviridis*	Calcium channels	[99]
VPS10 domain receptor protein SORCS 3–like	*L. rubripinnis*	Neuropeptide receptor	[99]
Lysosomal amannosidase	*L. rubripinnis*	Exoglycosidase	[99]
Ribonuclease-like 2	*L. rubripinnis*	Ribonuclease	[99]
KDR (kinase insert domain receptor)	*N. procyonoides*	Protooncogene, encoding a tyrosine kinase receptor	[105]
FPGT (Fucose-1-phosphate guanylyltransferase)	*Capreolus pygargus*	Guanylyltransferase	[98]
LRRIQ3 (Leucine-rich repeats and IQ motif containing 3)	*C. pygargus*	Protein-protein interaction	[98]
P-450 (Pda)	*Nectria haematococca*	Synthesis and breakdown (metabolism) of various molecules	[93,94]
GRMZM2G11056718 (genic sequence)	*Z. mays*	Protein binding	[102]
GRMZM2G013761 (genic sequence)	*Z. mays*	DEAD-box ATP-dependent RNA helicase 7	[102]
AF466202.2_FG007 (genic sequence)	*Z. mays*	Putative aldose reductase-related protein	[102]
GRMZM2G356653 (genic sequence)	*Z. mays*	Conserved mid region of cactin	[102]
CKAP2 (Cytoskeleton associated protein 2)	*E. plorans*	Cell cycling, and cell death	[78]
CAP-G (Capping actin protein, gelsolin)	*E. plorans*	Regulation of the mitochondrial ribosome assembly and of translational activity	[78]
MTG1(Mitochondrial ribosome associated GTPase 1)	*E. plorans*	Regulation of the mitochondrial ribosome assembly and of translational activity	[78]
HYI (Hydroxypyruvate isomerase)	*E. plorans*	Carbohydrate transport and metabolism	[78]
CIP2A (Cell proliferation regulating inhibitor of protein phosphatase 2A)	*E. plorans*	Anchorage-independent cell growth and tumor formation	[78]
KIF20A (Kinesin family member 20A)	*E. plorans*	Transport of Golgi membranes and associated vesicles along microtubules	[78]
MYCB2 (MYC binding protein 2)	*E. plorans*	Protein homodimerization activity and ligase activity	[78]
SLIT (Slit guidance ligand 1)	*E. plorans*	Calcium ion binding	[78]
TOP2A Topoisomerase (DNA) II alpha	*E. plorans*	Poly(A) RNA binding and protein heterodimerization activity	[78]
CAP-G (Capping actin protein, gelsolin)	*E. plorans*	Actin binding	[78]
GTPB6 (GTP binding protein 6)	*E. plorans*	Binding protein	[78]
(RTEL) 1-like Argonaute-like protein	*S. cereale*	Gene silencing by RNA	[77]
XRCC2	*A. latifasciata*	DNA repair protein	[25]
(SYCP) 2 Synaptonemal complex protein 2	*A. latifasciata*	DNA binding	[25]
(CENP) E Centromere-associated protein	*A. latifasciata*	Chromosome congression, microtubule-kinetochore conjugation and spindle assembly checkpoint activation	[25]
ESPL Separin-like	*A. latifasciata*	Chromosome segregation	[25]
Aurora kinase (AURK) A-B-like	*A. latifasciata*	Microtubule formation and/or stabilization at the spindle pole during chromosome segregation	[25]
Kinesin-like protein KIF11-like	*A. latifasciata*	Establishing a bipolar spindle during mitosis	[25]
Tubulin beta-5 (TUBB5) chain-like	*A. latifasciata*	Structural component of microtubules	[25]
Spindle and kinetochore-associated (SKA) protein 1	*A. latifasciata*	Chromosome segregation	[25]
(RTEL) Regulator of telomere elongation Helicase 1-like	*A. latifasciata*	Stability, protection and elongation of telomeres and interacts with proteins in the shelterin complex known to protect telomeres during DNA replication	[25]
(TUBB1) Tubulin beta-1 chain-like)	*A. latifasciata*	Microtubules formation	[25]
INCENP (Inner centromere protein)	*Anolis carolinensi*	Binding centromere proteins	[74]
SPIRE2 (Spire type actin nucleation factor 2)	*Anolis carolinensi*	Actin binding	[74]
Vrk1 (Vaccinia-related kinase gene)	*A. flavicollis Apodemus peninsulae*	Regulate cell proliferation	[106]
18S rDNA	*Lates calcarifer*	Codon recognition by tRNAs	[79]

## Supplementary Materials

The following are available online at http://www.mdpi.com/2073-4409/8/2/156/s1. Supplementary Dataset S1: A complete set of B-linked genes; Supplementary Datataset S2: B-linked genes with the descriptions retrieved from ENSEMBL database; Supplementary Dataset S3, GO enrichment of B-linked genes.
